# Supplementary material for: Are carbon emissions trading and green financial instruments synergistic? -Comprehensive quantitative research based on content analysis
Source: PLoS One. 2024 Mar 7;19(3):e0298601. doi: 10.1371/journal.pone.0298601 (PMC10919601; doi:10.1371/journal.pone.0298601)
Supplement: S1 Data — (ZIP) [file pone.0298601.s001.zip › Data/地级市Z_F工作报告.zip!/║■▒▒/╬Σ║║╩╨/└┤╫╘╣1⁄2╓┌║┼í╛┬φ┐╦╩2╛▌═°í┐_2015.docx]

各位代表：

现在，我代表市人民政府向大会报告工作，请予审议，并请市政协委员和其他列席人员提出意见。

一、2014年工作回顾

2014年是武汉全面深化改革，综合经济实力和城市功能提升取得重大突破的一年。我们在省委、省政府和市委的坚强领导下，认真学习贯彻习近平总书记系列重要讲话精神，深入落实市委的决策部署，坚持稳中求进的工作总基调和“竞进提质、升级增效”的总要求，践行“三维纲要”，紧紧围绕建设国家中心城市、复兴大武汉的目标，大力实施“五大计划”，沉着应对各种压力挑战，较好完成了市十三届人大三次会议确定的主要目标任务，经济社会发展跃上新台阶。

——经济总量突破万亿大关。地区生产总值提前一年完成“十二五”规划确定的过万亿元目标，这是全市人民期盼多年、奋斗多年，凝聚着城市光荣与梦想的历史性突破，是武汉发展的重要里程碑。地方一般公共预算收入迈上千亿台阶，达到1101亿元。全社会固定资产投资7002.85亿元，增长16.7%。社会消费品零售总额4369.32亿元，增长12.7%。

——先行先试一批改革创新举措。在全国率先推出规范行政权力运行的权力清单、程序清单和责任清单“三联单”制度，高校、科研机构职务科技成果使用、处置和收益管理改革走在全国前列。“青桐计划”点燃大学生创新创业激情，新增大学生创业企业超过600家。国务院明确武汉开展国家创新型城市试点。

——武汉三镇通地铁。轨道交通4号线二期开通试运营，与4号线一期、2号线一期，共同串联起汉口、汉阳、武昌，三镇同圆“地铁梦”，武汉交通格局正在改写。

——国际大通道网加快形成。新开通武汉至旧金山、莫斯科等国际及地区航线7条，累计达到31条；国际及地区旅客吞吐量130万人次，均居中部第一。对接国家“一带一路”战略，开通武汉至东盟四国的近洋航线，中欧(武汉)国际铁路货运班列实现常态化运营。

——城市环境显著改善。同步推进“三创”工作，掀起创建全国文明城市热潮，创建国家卫生城市通过国家技术评估，城市形象不断提升。

——文化新品牌亮点纷呈。首届WTA武汉网球公开赛成功举办。城市文化新地标汉秀剧场、万达电影乐园、光谷国际网球中心投入使用。“看武网、赏汉秀”成为城市新品牌。

——民生福祉不断增进。全市公共财政用于民生领域的支出达793.7亿元，增长9.8%，民生支出占公共财政支出比重达67.3%，比上年提高2.9个百分点，解决了一批涉及群众切身利益的居住、出行、教育、医疗、社保、食品药品安全等问题。城镇常住居民人均可支配收入增长9.9%；农村常住居民人均可支配收入增长12.3%。

武汉，每天不一样!武汉力量更加雄厚，武汉精神更加自觉，武汉自信更加坚定，武汉形象更加鲜明!勤劳智慧的武汉人民，正满怀豪情、奋勇前行在建设国家中心城市、复兴大武汉的伟大征程上!

过去的一年，我们主要抓了五个方面工作：

(一)稳增长，促转型，经济规模质量同步提升

强化工业支撑作用。深入实施工业倍增计划，坚持不懈抓工业投资、产业发展、重大项目和园区建设。完成工业投资2606亿元。上海通用武汉生产基地一期、北车轨道交通装备修造基地、周大福珠宝文化产业园等5个投资50亿元以上工业项目建成投产；开工建设武汉华星光电、东风雷诺、华为光电子生产研发基地等5个投资50亿元以上工业项目；新引进10个投资50亿元以上工业项目。“大光谷、大车都、大临空、大临港”四大工业板块建设扎实推进。工业倍增发展区新增开发面积50平方公里。支柱产业实力不断增强，汽车及零部件、电子信息、食品烟草、石油化工等产业保持快速增长，汽车产销量均突破百万辆。启动实施千企升级计划，完成技术改造投资650亿元，推进400户骨干企业改造升级。工业总产值突破1.3万亿元。

提升服务业规模水平。大力实施服务业升级计划。生产性服务业特色明显、业态优化。引进金融机构8家，多层次资本市场蓬勃发展，武汉民间金融街建成开街，金融机构各项贷款余额14463亿元，增长13%。社会物流总额突破2.5万亿元，获批两岸冷链物流产业合作试点城市，东西湖综合物流园成为首批国家级示范物流基地。举办各类展会708场次。工程设计产业产值、软件和信息服务业收入均突破1000亿元，成功入选“宽带中国”示范城市。武昌·长江文创设计产业园获批国家文化产业试验区，“汉阳造”文化创意产业园获批国家级广告创意园区。商贸流通现代化步伐加快。电子商务交易额突破3000亿元，增长68.5%；武汉宜家、武商众圆广场等10个大型商业综合体开业。成功创建全国旅游标准化示范城市，黄陂木兰文化生态旅游区获批国家5A级旅游景区；全市5A级景区达到3个，5家旅行社进入全国百强，均居全国副省级城市首位。引进投资30亿元以上现代服务业项目10个。

加大创新驱动发展力度。加快实施自主创新能力提升计划。推动出台《东湖国家自主创新示范区条例》、《武汉市知识产权促进和保护条例》。

科技体制改革深入推进。全面落实“黄金十条”。扩大知识产权融资、“萌芽贷”等科技信贷，新增科技贷款176亿元、科技保险207亿元。新增新三板挂牌企业43家，累计79家。各类创投机构330家，管理资本总量300亿元。建立武汉人才创新创业超市，探索实施外籍高层次人才“绿卡”制度，引进海内外高层次人才259名。

区域创新体系不断完善。支持10家工业技术研究院引进海内外研究团队140个，孵化科技型企业137家。国家级产业技术创新战略联盟和企业研发中心分别达到7家和23家。组织实施关键共性技术攻关项目73个。发明专利授权量3900件。技术合同交易额309亿元。

战略性新兴产业规模不断壮大。编制实施加快光电子信息、生物健康、新能源等优势产业集聚发展规划纲要。积极参与、推动设立总规模为300亿元的集成电路产业基金。引进武汉天马二期等一批重大项目。武汉未来科技城、光谷生物城等特色园区产业聚集度不断提升。新增高新技术企业163家，总数达1276家 完美世界小说。高新技术产业实现产值6725亿元，增长20%。

(二)重改革，扩开放，发展动力活力明显增强

深化经济体制改革。研究制定新一轮国资国企改革总体方案。完成武汉国资公司与武商联集团整合重组。完善公共财政体制，全口径预算管理有序推进。降低准入门槛，面向民间资本，推出38个总投资1012亿元招标项目。推进工商登记制度改革，全面实施注册资本“实缴改认缴”、“先照后证”等制度。市场活力进一步释放，新增各类市场主体13.68万户，增长26.4%。

加快政府职能转变。出台《武汉市政府职能转变和机构改革实施意见》。市级行政审批事项由259项减少到240项。市场监管、农业农村管理等领域机构调整有序推进，市、区、街(乡镇)三级食品药品监管机构基本建立。完成561家事业单位分类工作。推出政府向社会购买公共服务目录。加大政务公开力度，市、区全面公开财政预决算和“三公”经费预算信息。开展电视问政、民评民议活动，深化“治庸问责”，坚决查处行政不作为、乱作为、慢作为。坚持依法行政。组建市政府法律顾问团。出台《武汉市行政过错责任追究办法》。提请市人大常委会审议地方性法规草案5件，制定政府规章9件，人大议案、代表建议和政协建议案、提案1074件全部办结。

提升城市国际化水平。开放型经济加快发展，新引进世界500强企业16家，累计216家；实际利用外资62亿美元，增长18.1%；完成进出口总额264.29亿美元，增长21.4%；对外直接投资额8.25亿美元,增长3.8倍。开放功能进一步提升，中国内陆(湖北武汉)自由贸易试验区申报和先行先试积极推进；武汉外国领事馆区规划建设方案获国家批复；天河机场获批实行过境72小时免办签证政策，武汉成为过境免签城市；武汉经济技术开发区国际教育园区建成使用。国际交流合作广泛深入，协作开展了中法建交50周年系列纪念活动，开启法国巴黎中国文化中心“武汉之窗”；成功举办中俄万里茶道城市市长高峰论坛，与硅谷11个城市共同发布加强合作《双谷宣言》，新增28个国际友好交流城市。

(三)强基础，优环境，现代化大都市风貌日益彰显

强力推进城建攻坚。坚持市、区共建，城市基础设施建设投资达到1515亿元。城建重大项目竣工17个、开工17个、续建33个。

交通枢纽建设取得新进展。武汉至黄石(黄冈)城际铁路、江北铁路一期、机场高速公路建成通车。武汉长江中游航运中心建设全面展开，阳逻港集装箱吞吐量首次突破百万标箱，迈入世界内河集装箱港口第一方阵。天河机场T3航站楼建设冲出“地平线”，第二跑道及交通中心建设抓紧推进。

城市路网建设加快。鹦鹉洲长江大桥建成通车。三环线北段改造工程完工。长江大道一期、姑嫂树路快速通道、水果湖片区交通改善工程竣工通车。轨道交通加快向新城区延伸，1号线汉口北延长线开通试运营。轨道交通机场线、21号线、27号线、29号线和杨泗港长江大桥、沌口长江大桥等项目开工建设。轨道交通3号线一期、6号线一期、7号线一期、8号线一期，江汉六桥、东湖通道、东风大道、雄楚大街改造等重大项目加快推进。建成微循环道路54条、慢行交通系统63.5公里。

市政设施不断完善。新增排水干管100公里，疏浚管涵4956公里。龙王嘴污水处理厂改扩建工程建成投入运行。新增污水管网132公里。完成8个自来水厂饮用水源地隔离防护工程。建成地埋式垃圾转运站30座。城市排渍能力、污水收集处理能力、供水安全保障能力和垃圾处理能力有效提升。

城市更新改造有序开展。三环内“三旧”改造全面启动。完成11个城中村整体拆除。汉正街、二七片、龟北片、青山棚户区等重点片区改造加速实施。

深入推进“城管革命”。全面落实《武汉市城市综合管理条例》。集贸市场、小餐饮、食品小作坊、建筑工地、背街小巷、铁路沿线、违法建设等专项整治取得明显成效。深化城市管理执法体制和考评机制改革，稳步推进环卫作业市场化改革，城市综合管理效能进一步增强。

加强生态环境保护。“两型社会”建设综合配套改革试验取得新进展。制订《武汉市主体功能区规划》、《武汉市全域生态框架保护规划》，全面划定和严守生态保护红线。启动湿地自然保护区生态补偿试点。实施绿满江城行动计划。第十届园博会筹备工作扎实开展。张公堤城市森林公园绿带基本形成，沙湖公园、戴家湖公园基本建成。新建绿道222公里，新增绿地668万平方米。出台《武汉市山体保护办法》，完成6000亩破损山体生态修复。市域166个湖泊“三线一路”保护规划全面编制完成。开展国家水生态文明城市试点。实施改善空气质量行动计划，城市空气质量优良天数182天，比上年增加22天，PM2.5平均浓度比上年下降12.8%。中法武汉生态示范城启动建设。国家环保模范城市创建积极推进。

(四)抓统筹，兴新城，城乡一体化取得新进展

着力提升城镇化质量。制订实施《武汉市新型城镇化规划》。获批国家新型城镇化综合试点。武汉经济技术开发区与汉南区一体化管理体制机制基本形成。阳逻、吴家山、纸坊、常福、盘龙、纱帽等产业新城建设加快，落户投资5亿元以上工业项目32个。

大力发展现代都市农业。7万亩设施蔬菜基地建设成效初显，主要“菜篮子”产品综合自给率达73.5%。新增国家级农业标准化示范区2个。实现农产品加工产值2310亿元。赏花经济发展迅速。新型农业经营主体不断发展，新增家庭农场840个、农民合作社980个。农村产权改革确权登记任务全面完成。

完善农村基础设施。综合整治大型泵站主引水港5条，更新改造骨干泵站179座，除险加固病险水库30座，整治排灌港渠3286公里。中央投资农田水利建设年度项目全面完成。新建“村邮站”500个。

(五)惠民生，促和谐，社会建设全面加强

提升基本民生保障水平。深化全民创业，城镇新增就业18.64万人，转移农村劳动力5.68万人，扶持自主创业2.9万人。社会保险净增参保59.4万人次，城乡居民基本养老保险基础养老金提高20%。保障性住房新开工8.5万套，基本建成4.9万套，分配入住3.6万套。公租房、廉租房实现并轨管理。累计发放困难群众救助补贴20多亿元。新建改建社区养老院104家，新增城乡居家养老服务场地141处。获批全国养老服务业综合改革试点城市。

完善公共服务体系。各级各类教育全面发展。通过国家义务教育基本均衡督导评估。完成100所公办小学标准化学校建设任务。新增适龄幼儿入园学位9000个。高考全口径升学率保持在93%以上。教育云“三通两平台”建设基本完成。

国家医疗卫生服务中心建设加快。新增国家临床重点专科26个，总数达109个。新增三级医院6家，总数达45家，居全国城市第三位。完成30家基层医疗卫生机构提档升级，建成50个中心村卫生室。全科(家庭)医生、乡村医生签约服务200万人。

文化事业繁荣发展。“文化五城”建设扎实推进。公共图书服务网络不断健全。开展各类文化惠民活动2万多场。5项文艺精品获国际和国家奖项。启动建设武汉“中共中央机关旧址纪念馆”。成功举办武汉国际杂技节、男篮亚洲杯赛等大型文体活动。

推进社会治理创新。全面深化1350个幸福社区创建工作，社区组织协助政府服务事项由165项减至16项，被授予“全国和谐社区建设示范城市”。激发社会组织活力，新增社会组织2103家。长江救援志愿队用生命托起城市大爱，感人事迹享誉全国。“六五”普法扎实开展。信访工作制度改革有力推进。社会治安防控体系不断完善，全国深化平安建设现场会在武汉召开。食品药品监管、消防、安全生产工作进一步加强，杜绝了重特大安全事故发生。

协调发展各项社会事业。“单独两孩”政策平稳实施，出生人口性别比持续下降。争创第六届“全国双拥模范城市”、援疆援藏工作扎实推进。国家安全、国防动员、民防、仲裁、保密、参事、气象工作不断加强，文史、档案、民族、宗教、侨务、科协、地方志工作取得新成绩，妇女、儿童、老龄、残疾人事业健康发展。

市政府承诺的十件实事全面完成。

我们在努力完成年度目标任务的同时，着眼长远，积极做好落实武汉2049远景发展战略的相关工作。

各位代表，过去的一年，我市经济社会保持了持续健康发展，成绩来之不易。这是党中央、国务院亲切关怀的结果，是省委、省政府和市委正确领导的结果，是全市人民团结拼搏的结果。在此，我代表市人民政府，向辛勤工作在各个岗位上的全市各族人民，向给予政府工作大力支持的人大代表、政协委员，向各民主党派、工商联、各人民团体和社会各界人士，表示崇高的敬意!向中央在汉单位和各类驻汉机构，向驻汉部队、武警官兵，向所有关心支持武汉建设发展的港澳台同胞、海外侨胞和国际友人，表示衷心的感谢!

在肯定成绩的同时，我们清醒地认识到，加快发展仍然是武汉当前最重要的任务，新常态下，武汉既面临难得的发展新机遇，也会出现新情况、遇到新问题、面临新挑战。主要是：产业规模不够大，实力和竞争力还不够强，传统产业转型升级任务艰巨，新兴产业成为支柱尚需时日，继续保持中高速增长压力和困难增大；国有资本能量没有得到充分释放，民营经济发展相对滞后；长期积累的结构性矛盾和体制性障碍进一步凸显，改革攻坚任务相当艰巨；城市建设任务依然繁重，城市服务管理存在薄弱环节，交通拥堵、空气污染等“大城市病”依然突出，就业、社会保障、住房、教育、医疗、食品安全等民生工作离群众期望还有差距；多元化群体利益的协调难度增大，创新社会治理更加迫切；政府工作中还有缺点和不足，“庸懒散”问题依然存在。对于这些问题，我们将在今后工作中加倍努力，切实加以解决。二、2015年工作建议

各位代表，市委十二届六次全会确立了“万亿倍增”的新目标，提出用七年时间，实现经济总量由一万亿向两万亿跨越。2015年是向“万亿倍增”目标迈进的开局之年，是“十二五”规划的收官之年，是加快建设国家中心城市的重要一年。做好今年的政府工作，非常重要。

当前，武汉的发展已步入上升通道，我们虽然面临一些困难和挑战，但更具有跨越发展的有利条件。国家深入实施促进中部崛起、“一带一路”、长江经济带和创新驱动发展、扩大内需等战略，为处在战略交汇点上的武汉，提供了难得机遇，开辟了新的发展空间。

我们必须抓住机遇、乘势而上，迎接挑战、锐意进取，以义不容辞的责任担当、奋发有为的工作激情，把武汉的事情办得更好，把我们的城市发展得更好!

2015年政府工作的总体要求是：全面贯彻落实党的十八大和十八届三中、四中全会精神，深入学习贯彻习近平总书记系列重要讲话精神，积极把握和主动适应经济发展新常态，围绕“支点之点”，突出运用改革方法释放发展潜力，突出创新驱动加快转型升级，突出建管并重提升城市功能品质，突出完善公共服务增进民生福祉，突出保护生态环境建设美丽江城，突出严格依法行政建设法治政府，努力实现经济平稳较快增长，确保社会和谐稳定，圆满完成“十二五”规划的各项目标任务，为建设国家中心城市、复兴大武汉奠定坚实基础。

经济社会发展的主要预期目标是：地区生产总值增长10%左右；地方一般公共预算收入增长11%；全社会固定资产投资增长15%，社会消费品零售总额增长13%；城乡居民收入增长与经济增长同步。全面完成省下达的节能减排任务。

上述指标是我们在科学分析后确立的，是我们期望的发展目标，是增强信心、稳定人心的目标，更是提振全市干部群众精神的目标。我们立足于自我加压，“跳起来摘桃子”，就是要给市场一个明确的信号，增强各类市场主体在武汉发展的信心。在实际工作中，我们将坚持实事求是，确保实实在在的增长。

今年重点抓好以下七个方面工作：

(一)坚持工业强基、两业并举，加快产业结构优化升级

做大做强先进制造业，加快发展现代服务业，促进增量优质、存量优化，加快构建结构合理、优势突出的现代产业体系。

谋划推进“工业倍增升级版”。牢牢扭住工业这个牛鼻子，制订实施“武汉制造2025”行动计划。加快100项工业重大项目建设，工业投资突破3000亿元。确保东风雷诺、东风新能源汽车、格特拉克变速箱等5个投资50亿元以上项目建成投产；确保武汉天马二期、东本三厂、上海通用武汉生产基地二期、金发科技、圣德利薄膜太阳能电池、武桥海工装备基地、比亚迪新能源汽车等投资50亿元以上项目开工建设；确保武汉华星光电、长飞科技园、华为光电子生产研发基地等投资50亿元以上在建项目顺利实施。提升工业倍增发展区承载功能，加快四大工业板块建设。大力实施千企升级计划，重点推进300项技改项目。制定工业互联网发展路线图和行动方案，推动工业化和信息化深度融合。工业总产值达到1.5万亿元。

加快服务业向中高端发展。促进服务业加快融入互联网时代，大力发展研发、创意设计、电子商务、智能物流、互联网金融、信息服务等新业态。推进金融创新，引进金融机构8家以上，新增地方法人金融机构3家，推动8家企业在境内外上市，鼓励规范民间金融发展。发展现代物流，加快“一港六园八中心”建设，推进多式联运，开展城市共同配送试点。完善武汉国际博览中心整体配套功能，引进一批国内外知名展会。深化服务外包示范城市建设，努力创建中国软件名城。积极申报“世界工程设计之都”。支持传统商贸企业探索线上线下融合发展新模式，加快国家电子商务示范基地建设，电子商务交易额突破4000亿元。推进国家智慧旅游城市建设。全面启动江汉朝宗文化旅游区5A景区创建工作，实施两江四岸核心区趸船码头综合整治、景观亮化提升等工程。支持东湖风景区创建国家生态旅游示范区。突出高端定位、总部优先，加快建设武汉中央商务区、汉口沿江商务区、武汉中央文化区、汉正街·武汉中央服务区、武汉国际博览会展区、武昌滨江文化商务区、杨春湖高铁商务区、青山滨江商务区等服务业聚集区。支持汉口北国际商品交易中心申报开展“国家内外贸结合商品市场试点”。

(二)坚持改革引领、开放先导，增强经济社会发展新动力

改革开放是发展的活力源泉，是向更高水平迈进的强大动力。抓紧推出一批激活力、强动力、扩空间效用明显的改革开放举措，充分释放改革开放红利。

发挥经济体制改革的牵引作用。加大简政放权力度。建立权力清单动态调整机制。全面清理取消非行政许可审批事项，规范行政审批中介服务。完成新一轮政府机构改革，积极稳妥推进事业单位改革。

深化国有企业改革。加快国有企业混合所有制改革。积极探索国有企业股权激励及员工持股。完成经营性国有资产集中统一监管全覆盖，分行业组建或改组国有资本投资运营公司。

加快发展民营经济。拓宽民间资本投资领域，制订公共服务领域向民间资本开放计划。鼓励支持民营企业参与国有企业改革，切实保障民营企业合法权益。完善支持小微企业发展的政策措施。

深化财税体制改革。完善财政转移支付制度，强化专项资金使用监管。建立债务风险预警和应急处置机制。创新政府采购方式，探索实施通用类货物“商场供货(含电商)+网上竞价”采购。

完善现代市场体系。深入推进工商注册制度便利化改革，实行“宽进严管”，建立健全事中事后监管机制。健全社会征信体系、企业信用监管体系。有序推进非基本公共服务、资源、环保等领域价格改革。加快完善多层次资本市场体系，大力发展区域性场外交易市场。

拓展开放的广度和深度。努力建设内陆经济开放高地。把招商引资作为经济工作的重中之重，创新招商引资理念和模式，完善招商引资政策，充实招商引资重点目标企业库，强化招商引资责任制。各中心城区至少引进1个投资30亿元以上服务业项目，各开发区和新城区至少引进1个投资50亿元以上工业项目。实际利用外资增长18%以上，招商引资总额增长30%以上。实施外贸转型工程，外贸出口增长6%以上。鼓励支持优势企业“走出去”发展。加强与港澳台经贸文化交流合作。

提升对外开放功能。拓展国际大通道，新开国际及地区航线3条；开通武汉至日韩近洋航班；提升中欧(武汉)国际铁路货运班列双向常态化运营水平。力争获批武汉新港空港综合保税区，提升铁路口岸开放功能。加快中国内陆(湖北武汉)自由贸易试验区申报和先行先试工作。

加强国际交流合作平台建设。加快外国领事馆区建设，做好英国领事馆开馆服务和美国领事馆开办签证协调工作。推动中法武汉生态示范城建设。新增国际友好城市和友好交流城市10个以上。扩大“双谷双城”合作，加强与俄罗斯伏尔加河沿岸联邦区城市的交流互动。深入实施外籍人士“家在武汉”工程，推进国际化示范街区建设。

深度融入长江经济带建设。制定贯彻落实国务院长江经济带发展指导意见的实施意见。加快武汉长江中游航运中心建设，完善阳逻港集疏运体系，深化与长江流域城市航运合作。大力发展临空临港产业。积极参与长江中游城市群建设发展。深入落实全省“两圈两带”发展战略。

(三)坚持创新驱动、转型发展，全方位、全体系、全区域、全领域建设创新型城市

把建设国家创新型城市放在武汉发展全局的核心位置，以全球视野、战略思维谋划创新，用最大的热情、最好的资源、最优的政策、最多的投入推动创新，为建设国家中心城市提供强大动力支撑。

加快构建有利于创新驱动发展的体制机制。认真实施《东湖国家自主创新示范区条例》和《东湖高新区先行先试实施方案》，充分发挥示范区创新引擎作用。完善落实“一区多园”机制，推动示范区先行先试政策在全市开花结果。全面推进职务科技成果使用、处置和收益管理改革，促进成果转化。全方位改革政府科技资金管理、计划项目管理体制机制。加快打造科技金融特区、人才特区。新增科技贷款余额150亿元、科技保险保额200亿元；新引进创投机构30家。深入实施“黄鹤英才计划”、“3551光谷人才计划”，探索人才与风险投资结合机制。积极支持和引进院士工作站，充分发挥院士、专家在创新创造中的重要作用。

加快构筑以企业为主体的技术创新平台体系。制定出台鼓励支持企业自主创新的意见，充分发挥企业在技术创新决策、研发投入、科研组织和成果转化中的主体作用。实施大中型企业研发机构全覆盖行动，鼓励企业与高校院所联合建设国内一流、国际先进的技术研发平台。引进一批世界500强企业研发机构。推进应用型技术研发机构市场化、企业化改制。向企业发放科技创新券，激发企业创新活力。加快发展一批工业技术研究院、产业技术创新战略联盟、技术标准战略联盟等创新创业平台，对接产业需求，研发和转化一批科技成果。新增科技企业孵化器面积100万平方米。高标准建设国家知识产权示范城市。

加快发展战略性新兴产业。围绕产业链部署创新链，把创新成果变成实实在在的产业活动，培育形成新的增长点。大力发展光电子信息、生物健康等优势产业，壮大节能环保、高端装备制造产业规模。按照千亿目标，聚焦发展集成电路、新能源汽车、工业机器人、大数据、北斗导航等新兴产业，使新兴产业成为“万亿倍增”的重要支撑。进一步扩大武汉未来科技城、光谷生物城等特色园区产业集聚规模，推动各开发区、各区积极创建科技园区，依托园区培育和引进更多高新技术企业。深入实施科技型中小企业成长路线图计划，新增高新技术企业100家 礼拜五秘书网。高新技术产业产值突破8000亿元，增长20%。

加快营造有利于创新创业的环境。深入实施“青桐计划”，充分激发百万大学生创业潜能。新增大学生创业特区15个，吸纳10万名大学生创新创业。加快建设一批廉租办公室、创业工坊和人才公寓，完善创新创业配套服务体系。广泛开展创新型机关、园区、街道、社区创建，把“创新”镌刻在武汉的各行各业、每个角落。落实全民科学素质纲要，营造崇尚创新、鼓励创业、宽容失败的社会氛围，使创新成为城市之魂。

(四)坚持建管并重、品质为先，着力提升城市功能和宜居水平

遵循特大中心城市建设规律，更新城建理念，更加注重城市品质，更加关注市民意愿，完善基础设施，提升管理水平，改善生态环境，努力让城市安静下来，努力让市民在家门口享受大自然。

强化规划先导作用。启动修编新一轮城市总体规划、土地利用总体规划、综合交通规划。推进市域城乡规划和土地利用规划“两规合一”，实现“一张图”管理。

持续高质量推进城市建设。坚持投入不减、力度不减，加快构建现代化、高品质的基础设施体系。城市基础设施投资达到1750亿元。

创新城建投融资体制。完善市、区共建体制，发挥市、区两个积极性。积极推广政府和社会资本合作模式(PPP)。通过特许经营、投资补助、政府购买服务等方式，鼓励引导社会资本参与基础设施建设。推动优质资产上市融资，增强平台公司资本化运作能力。

巩固提升交通枢纽功能。主动融入长江经济带综合立体交通走廊建设。加快推进天河机场三期、阳逻港集装箱三期、武深高速武汉段、汉孝大道武汉段、硚孝高速武汉段、四环线等重大项目建设。积极推动沿江高铁、武西高铁规划建设。支持武汉至孝感城际铁路开通运营。

完善市域快速交通系统。坚持以轨道交通引领城市发展格局，牢记“地铁生日”，确保3号线一期建成通车，实现中心城区地铁初步成网；精心组织已开工地铁线路建设，开工建设1号线径河延长线和24号线。加快过江通道、城市环线和快速路建设。建成东湖通道、江汉六桥、三官汉江公路大桥。加快建设临空港区跨府河大桥。实现二环线全线贯通。完成三环线、东风大道、雄楚大街和21号公路改造工程。开工建设青山长江大桥、岱家山大桥、巨龙大道延长线。实施汉阳大道、中山大道景观提升工程。

切实抓好“绿道年”、“路网年”、“停车场年”建设。中心城区，武昌地区重点建设环东湖绿道；汉口地区重点建设张公堤绿道，连接汉江绿道、汉口江滩，形成串联汉口主要区域的绿环；汉阳地区重点建设环墨水湖绿道。新城区，重点建设环后官湖绿道、环青龙山绿道、环金银湖绿道与府河湿地绿道、木兰景区群绿道。全市新建绿道228公里。围绕改善提升重点功能区、大型生活区等区域交通条件，打通断头路、畅通微循环，改造连通道60条，形成对接快速路的次支路网；制订规划，出台政策，多途并举，加快配建一批停车场，增加停车位；严格交通管理，力争每个区消除4-5个堵点，使城市拥堵状况明显改观。坚持公交优先战略，对接轨道交通，优化公交线网，加快形成“快、干、支、微”的公交线网新格局，让地面公共交通快起来。

加强市政基础设施建设。建成阳逻自来水厂二期工程，完成余家头自来水厂改扩建、琴断口自来水厂升级工程，启动白鹤嘴、宗关自来水厂改扩建。新建排水干管100公里。启动北湖污水处理厂及配套污水深隧工程建设，推进南太子湖污水处理厂升级改造、黄家湖污水处理厂改扩建项目。新增污水管网100公里。建成长山口垃圾焚烧厂二期工程。加快建设现代化智能电网。建成天然气高压外环线，构建全市天然气调度“一张网”。

加快推进“三旧”改造。整体拆除城中村10个以上，还建房项目开工、竣工各300万平方米以上。各中心城区完成50万平方米以上的旧城、旧厂征收任务，启动两片以上棚户区改造。逐步消除D级危房，确保居民住房安全。

各位代表，持续大规模的城市建设，给市民的生活与出行带来不便和影响，广大市民给予了充分理解和大力支持，与我们一道，共担压力、共建家园。我们将坚持以人为本，调整重点、把握节奏、优化时序、强化管理，快完工、慎开工，尽量缩短建设施工影响市民出行的“阵痛期”，让广大市民尽早享受到快速路网的便捷，更多享受到城市功能品质提升带来的美好生活!

提高城市管理水平，促进精神文明建设。升华理念、提升标准、完善制度，巩固提升“城管革命”成果。理顺城管执法体制，在街道层面探索城市综合管理机制改革。加快推进环卫作业、绿化养护市场化。全面提升主次干道、窗口地带保洁水平。强化铁路沿线、背街小巷、城乡接合部等薄弱区域环境综合治理。强化物业服务质量监管，破解住宅小区综合管理难题。深入开展“三创”工作，力争国家卫生城市、全国文明城市、省环保模范城市创建成功，并建立长效机制，巩固创建成果。

治城育人，弘扬社会主义核心价值观，形成催生“武汉群星”现象的常态机制，让城市明星在武汉有地位、载入史册，激励广大市民学习、争做模范市民、功勋市民，不断增强城市认同感、荣誉感、自豪感。

大力推进生态文明建设。保护绿水青山，让城乡环境更美。深入实施绿满江城行动计划。加快建设三环线生态带，启动建设四环线生态带示范段、机场高速公路生态廊道。继续推进两江四岸江滩建设，建成武青堤江滩一期。基本建成龙灵山生态公园。全面完成破损山体生态修复。造林绿化10.6万亩。

全面开展水生态文明城市试点。深入实施水生态修复工程。严格落实湖泊“三线一路”保护规划，加快建设临江环湖沿河公园。加强沉湖、涨渡湖等湿地自然保护区建设，维护湿地原生态。切实保护长江、汉江、梁子湖等战略水源地安全。研究制订《武汉市水污染防治规划和行动计划》。

大力实施改善空气质量行动计划。重点治理PM2.5和PM10。积极参与武汉城市圈大气污染联防联控联治专项行动。加快推进国家新能源汽车示范推广试点工作。

全力办好第十届中国(武汉)国际园林博览会。按照“生态园博、绿色生活”的理念，加快建成园博园，确保园博会如期开园、精彩开幕，打造“百年精品、世纪园博”。(五)坚持统筹城乡、四化同步，加快推进新城区“独立成市”

加大“三农”工作力度，走以人为本、优化布局、生态文明、传承文化的新型城镇化之路，推进新城区产业、功能和城市格局现代化。

大力推进现代化新城建设。编制产城融合发展规划，推动产业项目向新城聚集。加快建设城镇市政道路、供水排水、污水处理、垃圾处理等基础设施，完善教育、医疗、文化等公共服务设施。积极开展生态乡镇、生态村“四级联创”，创建一批国家级“美丽乡村”、省级“宜居村庄”。

提升现代都市农业发展水平。推进10万亩设施蔬菜基地提档升级。探索“菜篮子”工程跨区域发展，在市外合作共建养殖基地。启动创建国家现代农业示范区，加快13个现代都市农业示范园区建设。新增农业标准化生产面积5万亩。建设武汉国家级种子产业园。发展农产品精深加工，实现产值2500亿元。高水平建设六大山水田园赏花区，推进赏花经济产业化、规模化、品牌化。

持续改善农村生产生活条件。实施41座小型水库除险加固，更新改造134处骨干泵站，整治3500公里排灌港渠，确保水利工程质量。加快农村电网改造。支持革命老区建设发展。

纵深推进农村改革。引导农村土地经营权有序流转，开展新一轮农村土地承包经营权确权颁证，推行土地流转风险保证金制度。鼓励规范工商资本进入农业领域，发展农业适度规模经营。提高农村资源资本化水平。积极发展农民股份合作。稳妥推进农村土地经营权、水域滩涂养殖权和农业设施抵押贷款。培育壮大新型农业经营主体，新增农民专业合作社300个、家庭农场600家。

(六)坚持为民惠民、共建共享，着力提升人民群众福祉

民生决定目的。我们要带着感情和责任，真诚倾听群众呼声，真实反映群众愿望，不断加大民生投入，着力解决涉及群众切身利益的民生问题，当好群众的贴心人、“圆梦人”，让市民生活年年都有新变化，一年更比一年好!

强化就业和社会保障工作。建立发展经济与扩大就业的联动机制，以创业兴业带动就业。城镇新增就业16万人，农村劳动力转移就业5万人，新增创业2.5万人，创业带动就业11万人。出台城乡居民基本养老保险配套政策，并与职工基本养老保险制度相衔接。改革社保业务办理方式，新建一批社保经办服务点，探索推进资源共享、全市通办。启动机关事业单位养老保险制度改革。集中解决一批困难群众的社保问题。社会保险净增参保35万人次，综合覆盖率保持在98%以上。确保完成省下达的住房保障目标任务。深化社会救助体系建设，完善临时救助制度，保障困难群众基本生活。抓好全国养老服务业综合改革试点。加快构建以居家为基础、社区为依托、机构为支撑的养老服务体系。

推进优质教育均等化。深化教育领域综合改革。继续实施新一轮学前教育三年行动计划。推行学区制、托管制，建立教育联盟和学校共同体，深化小学小班化试点，提高义务教育均衡优质发展水平。推动高中教育优质多样化发展。建立“年度教师”评选制度。推进产教融合、校企合作，扩大职业教育集团化办学覆盖面。支持武汉大学、华中科技大学等高校创建世界一流大学，支持江汉大学、武汉商学院等院校办出特色，努力建设一流的大学之城。

加快建设国家医疗卫生服务中心。加强“名医、名科、名院、名中心”建设。深化医药卫生体制改革，认真开展分级诊疗试点，分类实施公立医院改革，积极鼓励社会资本办医，新办高水平国际性医院取得实质性进展。努力创建全国基层中医药服务先进市。推进医养融合，实现中心城区居家养老医疗服务全覆盖。开工建设市老年病医院。认真实施预防保健强基工程。强化出生人口性别比综合治理，夯实计划生育基层基础工作。

提升城市文化品位。加快“文化五城”建设。大力推进文化惠民，实施实地观看各类演出观众突破1000万人次、服务读者突破1000万人次、1000万人次走进博物馆等“三个千万人次工程”。深化文化体制改革，进一步激发文化创造活力。支持创作一批思想精深、艺术精湛、制作精良的文艺精品。深度挖掘城市历史文化资源，建成武汉“中共中央机关旧址纪念馆”，加快建设盘龙城国家遗址考古公园，加强历史风貌街区保护与利用，做好万里茶道文物保护和申遗工作。广泛开展全民健身运动，促进体育产业发展。办好WTA武汉网球公开赛、世界中学生田径锦标赛、亚洲田径锦标赛等大型赛事。持续推进国家足球试点城市建设。

加强和创新社会治理。坚持依法治理，深化法治宣传教育，增强市民法治观念；畅通群众利益协调、权益保障法律渠道；把信访纳入法治化轨道；完善社区律师制度，加强法律援助工作。坚持系统治理，推进“全国和谐社区示范城市”建设；培育社会组织1800家以上。以网格化管理、社会化服务为方向，健全基层综合服务管理平台。依法加强民族宗教事务工作。深化平安武汉建设。创新发展具有武汉特色的立体化社会治安防控体系。依法治理网络空间，加强网络管控服务。牢固树立安全发展理念，切实抓好安全生产和人员密集场所安全管理薄弱问题、突出问题整改，有效防止和坚决遏制各类重特大事故发生。加强食品药品安全监管，积极创建国家食品安全城市。强化国防动员和国防教育，推进军民融合深度发展。

今年，我们将继续办好十件实事。

1、缓解市民出行难。开通微循环公交线路30条，增加公交车辆400台。建设30条、120公里公交专用道。改造提升三环线内道路交通标志标识、信号灯、护栏和安全防护等管理设施。建设公共停车位13500个。修复老旧破损人行道15万平方米。开展新一轮“万名干部进万村惠万民”活动，实现村村通客车。

2、改善就学就医条件。新建、完善300所中小学校内安全视频系统。在全市中小学安装学生直饮水系统。继续为新入学的全日制大学生每人赠送一本市民手册和一张100元的“武汉通”公交乘车卡。提档升级10家社区卫生服务中心、10家乡镇卫生院。启动订单式培养100名社区全科医生。为全市6万名新生儿实施免费耳聋基因筛查。

3、加强扶残济困。为一户双(多)残低保家庭发放护理补贴。对距离办理国家规定退休手续五年内的已参加城镇企业职工基本养老保险并正常缴费的灵活就业残疾人给予补贴。为本市户籍14周岁以下患白血病、重型再生障碍性贫血、脑瘫等重大疾病，且家庭贫困无力继续承担后续住院治疗费用的儿童，一次性提供5万元以内的临时大病医疗救助。

4、实施文体惠民。为2122个农家书屋补充更新出版物。在三镇选择35家游泳场所，为中小学生提供20天免费暑期游泳。对3500名青少年开展游泳、足球、网球免费培训。更新农村健身器材300套。

5、便利群众生活。新改扩建公共厕所100座。建设84座地埋式垃圾收集站。新建社区蔬菜便民店100家。为流动人口免费发放30万张“武汉通”式居住证。连片整治265个社区、60条城市道路架空管线容貌。在全市人员密集场所设置“出入境24小时自助服务机”69个。

6、改善空气质量。安装工地扬尘污染在线监控设施80套。推动100个建设工程工地安装喷洒降尘设施。淘汰7万辆黄标车和老旧车。从今年5月1日起，市域内全面禁止露天焚烧秸秆。

7、改善老旧社区基础设施和服务。加强100个老旧社区“幸福社区”创建工作，重点完善社区道路、排水、公共照明等基础设施。推进200个老旧社区绿化提档升级。续建100个社区消防室。

8、强化食品安全保障。在校园周边、集贸市场周边、老旧社区和城乡接合部，规范、设立小餐饮便民服务区36个。在白沙洲、四季美、皇经堂三大批发市场建设食品安全监督检测站。加强中小学、幼儿园食堂食品安全监管，学校食堂食品安全量化分级A、B等级达到65%以上。开展食品安全抽样检测3万批次，双月公布抽检信息。

9、完善养老服务。全市城乡居民基本养老保险基础养老金由每人每月144元增加到180元。提档升级30家社区养老院。新建社区老年人服务中心50家、农村老年人互助照料活动中心40家。

10、关爱农村留守儿童。新建10个留守儿童关爱服务站，实现留守儿童关爱全覆盖。举办面向留守儿童的家庭教育报告会50场、剧目演出20场。

各位代表，科学编制好“十三五”规划是今年的一项重要任务。我们要围绕加快建设国家中心城市、复兴大武汉的宏伟目标，切实谋划好推进“万亿倍增”、创新驱动等事关武汉当前和长远发展的各项工作，充分凝聚各方智慧、集思广益，编制好“十三五”规划，使之成为科学指导全市经济社会发展的宏伟蓝图和行动纲领。

(七)坚持依法行政、严格执法，加快建设法治政府

牢固树立法治理念，全面实施《武汉市法治政府建设规划》，不断提高运用法治思维和法治方式治理城市的能力和水平。

完善行政决策机制。把公众参与、专家论证、风险评估、合法性审查、集体讨论决定，明确为重大行政决策的法定程序，确保决策程序正当、过程公开、责任明确。制定出台《武汉市人民政府法律顾问工作规则》，实现各区(功能区)、各政府部门及街(乡镇)法律顾问全覆盖。推进新型智库建设，完善决策咨询制度。

深入推进依法行政。完善制度，推进机构、职能、权限、程序、责任法定化，切实做到“法无授权不可为、法定职责必须为”。深化行政执法体制改革，进一步推进相对集中行政处罚权工作，积极探索交通领域综合执法改革 金庸小说。加强对行使行政强制自由裁量权的监督检查，做到严格规范公正文明执法。支持审判机关和检察机关依法独立行使审判权和检察权。

强化权力运行制约监督。自觉接受市人大及其常委会的法律监督、工作监督和市政协的民主监督，切实办理好人大议案、建议和政协建议案、提案。自觉接受群众监督和舆论监督。健全行政监督体系，建立常态化监督制度，强化审计监督。深化政务公开，让权力在阳光下运行。

切实做到清廉为官、事业有为。深化“治庸问责”，扎实开展十大突出问题整改，坚决克服懒政、怠政，坚决惩处失职、渎职行为。切实履行政府党组党风廉政建设主体责任，认真落实“一岗双责”，全面推进惩治和预防腐败体系建设。严格落实中央八项规定、国务院约法三章、省委六条意见和市十一条禁令。抓好公务用车制度改革。建立健全反“四风”长效机制，巩固和拓展党的群众路线教育实践活动成果。

做好今年政府各项工作，责任重大。全体政府工作人员务必自觉践行习近平总书记提出的“三严三实”、“四有”要求，把心思集中在“想干事”上，把精力倾注在“能干事”上，把本领体现在“会干事”上，把目标锁定在“干成事”上，奋发有为激情干，求真务实科学干，敢于担当带头干，只争朝夕抓紧干，守规矩、有作为，努力创造经得起实践、人民、历史检验的实绩，和全市人民一道，让武汉每天不一样!

各位代表：

新的一年开启新的希望，新的征程承载新的梦想。实现“万亿倍增”、建设国家中心城市、复兴大武汉的光荣使命，鼓舞我们砥砺奋进；全市人民的信任期盼，鞭策我们勇往直前。让我们紧密团结在以习近平同志为总书记的党中央周围，在省委、省政府和市委的坚强领导下，敢为人先，追求卓越，谱写武汉改革开放和现代化建设新篇章!
